# Health and life insurance as an alternative to malpractice tort law

**DOI:** 10.1186/1472-6963-10-150

**Published:** 2010-06-02

**Authors:** Walton Sumner

**Affiliations:** 1 Department of Medicine, Washington University School of Medicine, St. Louis, Missouri, USA

## Abstract

**Background:**

Tort law has legitimate social purposes of deterrence, punishment and compensation, but medical tort law does none of these well. Tort law could be counterproductive in medicine, encouraging costly defensive practices that harm some patients, restricting access to care in some settings and discouraging innovation.

**Discussion:**

Patients might be better served by purchasing combined health and life insurance policies and waiving their right to pursue malpractice claims. The combined policy should encourage the insurer to profit by inexpensively delaying policyholders' deaths. A health and life insurer would attempt to minimize mortal risks to policyholders from any cause, including medical mistakes and could therefore pursue systematic quality improvement efforts. If policyholders trust the insurer to seek, develop and reward genuinely effective care; identify, deter and remediate poor care; and compensate survivors through the no-fault process of paying life insurance benefits, then tort law is largely redundant and the right to sue may be waived. If expensive defensive medicine can be avoided, that savings alone could pay for fairly large life insurance policies.

**Summary:**

Insurers are maligned largely because of their logical response to incentives that are misaligned with the interests of patients and physicians in the United States. Patient, provider and insurer incentives could be realigned by combining health and life insurance, allowing the insurer to use its considerable information access and analytic power to improve patient care. This arrangement would address the social goals of malpractice torts, so that policyholders could rationally waive their right to sue.

## Background

Health care is a risky endeavor [1]. The industry has made some progress in improving safety following the Institute of Medicine report, "To err is human", but malpractice reforms to reduce blame and improve accountability have lagged consistently [2].

Malpractice tort law has three theoretically important purposes [3]. First, threats of malpractice suits should deter physicians from practicing beyond their expertise. Second, malpractice suits should punish those who practice low quality care. Third, malpractice awards are a process for compensating patients injured by low quality care. One might hope that by achieving these purposes, malpractice tort law would further the broader goals of better health care and outcomes.

Unfortunately, current malpractice tort law does none of these tasks very well. Malpractice suits are not sensitive: real injuries are common but seldom pursued and only a small fraction result in paid claims [4-10]. This is in part due to the difficulty of detecting negligence: patients may file suits just to learn whether negligence occurred [11]. Malpractice suits are not specific: many invalid claims are paid and may be paid in proportion to the injury suffered rather than the quality of care delivered [9]. A recent review described a "good correlation" between the strength of evidence for medical negligence and physicians' probability of losing a malpractice trial [12], but correlation is not accuracy. Physicians lost 10-20% of cases with weak evidence of negligence, while patients won only 50% of cases with strong evidence of negligence. Malpractice claim history is predictive of future malpractice claims [13,14], but such claims largely reflect poor interpersonal skills [15-17]. High quality training may reduce risk [18] but in some cases the most knowledgeable physicians have been more likely to be sued than their peers [19]. In one specialty, board certified physicians experienced fewer state disciplinary actions than non-certified physicians, but equal numbers of malpractice claims [20]. It is therefore not at all clear that malpractice tort law compensates victims of medical mistakes or identifies clinically incompetent physicians.

Furthermore, adverse events are about equally frequent in malpractice tort systems and less expensive "no fault" systems for compensating disabled patients [21,22]. These observations suggest that malpractice tort law does no better at preventing mistakes or compensating victims than alternative systems.

Malpractice tort law has important unintended effects. Believing that they face a capricious threat of losing time, money and reputation, many physicians practice expensive defensive medicine [23-27]. The annual cost of defensive medicine in the United States is substantial, possibly as high as $100 billion [26,28-30]. Defensive medicine can be harmful as well [25,31]. For instance, all x-ray images entail at least a small risk of causing cancer [32]. Collectively, defensive radiographic imaging could pose a non-trivial public health problem [33]. False positive test results can initiate an injurious and expensive cascade of events. Still worse, high risk of malpractice suits may discourage technically qualified physicians from offering services that would benefit patients [25,34-37]. The unintended consequences of malpractice torts could cause harm without offsetting benefits to society.

Constructive change has been hard to achieve. A small survey reported that physicians do not believe that lawyers have the moral authority to guide medical practice and would prefer physician-led quality control measures [38]. Deliberate quality control efforts can reduce harmful variation in practice, decrease adverse events and prevent litigation [39-41]. In a particularly dramatic example, application of quality control methods to the very complex problem of ventilator management for adult respiratory distress syndrome (ARDS) patients improved their survival from less than 10% to more than 40%. The improvement occurred before the investigators learned anything new about adjusting ventilator settings for ARDS patients [40]. This is evidence-based medicine at its best: not an artificially constrained trial, but an exhaustive effort by dedicated clinicians informed by comprehensive observational data collection and analysis to make sense of the consequences of their actions for a wide range of patients. It was possible, at least in principle, for any hospital to deliver comparable care to ARDS patients, but only one group of researchers perceived a need to standardize a complex and variable health care process as a prerequisite for their studies. Physician-led quality control can be very effective, but is rare and usually addresses less complex problems than ARDS.

No-fault compensation for poor medical outcomes and independent arbitration are less confrontational and potentially less expensive complements to malpractice tort law. Apology laws have been advocated as another means of indirectly reducing medical error, ideally in combination with no-fault compensation [42]. However, United States citizens cannot be forced to accept these processes in place of malpractice torts, because the seventh amendment to the US Constitution guarantees trials by jury when sums of $20 or more are in question. The right can be abridged by legislative acts only if the justice system agrees that a compelling argument favors the abridgement. Therefore, US citizens would have to be persuaded by some compelling argument to waive this right. Guaranteed compensation would help, but it is not clear how strongly this would encourage general improvements in the quality of medical outcomes unless health care insurers provide the guarantee.

Physicians often call for caps on malpractice awards [43]. This may reduce malpractice insurance costs and even state health care costs [44], although a recent analysis suggests no effect [45]. Some argue that caps are not nearly ambitious enough reforms and call for a new system of medical justice [46,47], but sweeping reform is unlikely [43]. Other countries restrain malpractice activity by avoiding jury trials and contingency fees, restricting pretrial discovery and forcing the loser to pay the legal costs of the winner [48]. Caps and stronger reforms leave physician reputations at risk and the prospect of defending a suit remains daunting. The path of least resistance still may be to charge a patient, or an insurance pool, for a test that suggests that the physician left no stone unturned in the effort to serve the patient, even if the test has negative expected value and drains the insurance pool. Physicians facing any serious threat of malpractice suits are likely to feel similar pressure. Furthermore, these reforms do not provide direct incentives to improve patient care.

The Obama administration has rejected major tort reform as a component of federal health care reform. However, the administration has acknowledged that state experiments in tort reform could be instructive. The following discussion describes a possible insurance reform that could be implemented by states and how it might obviate medical tort law.

## Discussion

### Health and Life Insurance

There is only one group in the US health care industry that routinely collects enough data to monitor, compare and reward quality care. That group is not patients, physicians, pharmaceutical companies, public health agencies, hospitals, academics, or politicians. It is insurers. That every other group views insurers with deep and largely justified distrust is a tragic result of misaligned incentives: insurers increase profits by collecting premiums and not paying claims. Insurers must maintain a sufficiently respectable reputation to collect premiums from policyholders and must abide by contracts. A tense relationship with health care providers and patients is not surprising.

Combining health and life insurance in a single long-term policy should realign insurers' incentives and lend credibility to many insurer decisions [49,50]. Policyholders would pay insurance premiums for both health and life insurance, while the insurer would reimburse medical care. When a policyholder died, the insurer would pay a death benefit to the policyholder's designated beneficiaries. Neither the insurer nor the policyholder could casually terminate the relationship. Policies that endure a minimum of 5 years would give insurers the opportunity to reap some short-term reward for influencing health.

Policyholders could use a combination of term and permanent life insurance to convey longevity preferences to the insurer. Term policies convey interest in avoiding fatal accidents, while permanent policies (variable and whole life insurance) imply interest in longevity. Ideally, a basic policy would be established relatively early in life, with the expectation that policyholders could negotiate amendments to the life insurance policy. As in any viable insurance program, policyholders would need to initiate coverage while healthy, or pay a premium to purchase coverage with a pre-existing condition.

The insurer could profit in two ways. First, it could keep aggregate spending on health care below the sum of collections of health insurance premiums. Second, it could earn more from invested life insurance premiums than it pays in life insurance benefits if it can increase average life expectancy, whether by preventing accidental deaths, increasing longevity, or both. If the insurer finds inexpensive ways to heal, maintain health and extend longevity, the policyholders benefit. Conversely, neglect and other health care mistakes are likely to manifest as losses on life insurance and increased long-term health costs. This should create a tense resource allocation decision. It needs to be shared, as much as possible, between suffering patients, harried physicians and well-informed, highly analytical insurers.

The insurer could take a very broad perspective regarding policyholders' health. Profitable activities could be as diverse as lobbying against anti-health interests, subsidizing healthy behaviors and reducing environmental risks. A health and life insurer should have an especially strong interest in the quality of medical care provided to policyholders. In many situations, the insurer could profit twice from directing policyholders to high quality care, once if the care succeeds in avoiding complications and disability that would create additional medical expenses and again if the care reduces mortal risk. Conversely, the insurer would pay twice for failure to obtain high quality care for the average policyholder. Therefore, the insurer could reimburse health care systems or even individual physicians based on the quality of the care they deliver.

Health and life insurers would have incentives to develop nuanced measures of the quality of care. Pay for performance initiatives typically use simple quality measures and may not change outcomes appreciably [51-56]. Simple quality measures, such as the fraction of diabetics whose hemoglobin A1c is below 7%, could encourage physicians to avoid or drop patients who do not meet the precise goal [57]. Simple quality measures also are likely to focus too much attention on marginal improvements that are not cost-effective with respect to health. For instance, a provider might have greater financial incentive to help five patients reduce their hemoglobin A1c from 7.1% to 7% than to invest effort in a difficult patient at 10%. In contrast, a health and life insurer should reward providers who manage to reduce challenging diabetics' hemoglobin A1c from 10% to 8%, because this reduces long-term risk substantially. This is an example of a medical version of economists' "law of diminishing returns" and likely applies to every numeric goal in medicine: the greatest risk reduction occurs as extreme values move toward a more normal goal, not when the goal is reached.

The health and life insurer also should maintain a healthy skepticism regarding extrapolated quality measures. For instance, anticoagulation to prevent blood clots is clearly valuable for many orthopedic surgery patients, but involves serious trade-offs and may not change short-term mortality among internal medicine inpatients [58]. Nevertheless, thromboprophylaxis is a common quality measure in both orthopedic and medical settings. A health and life insurer could reasonably and credibly reject thromboprophylaxis as a quality measure in medical settings, or develop a nuanced policy identifying subsets of medical inpatients who do benefit from thromboprophylaxis. A similar analysis could address the recent controversy regarding breast cancer screening among women under age 50 [59]. Interestingly, the insurer might reject or endorse routine early mammography based largely on policyholders' life insurance purchases.

These calculations suggest a reimbursement strategy that could be called "fee for expected benefit" [60]. A health and life insurer has an opportunity to divide the profits accrued by delaying deaths, avoiding complications and reducing unnecessary care among providers and patients. The policyholder's life insurance purchases, the diseases suffered and providers' skill determine the treatment strategy and the profit that could be shared. The insurer could tailor these reimbursements to reinforce the careful teamwork required to achieve significant benefits for patients [61]. Most importantly, a health and life insurer paying fees for expected benefits should gain the credibility needed to reduce or deny payment for useless or overly expensive treatments - if the service could save costs by reducing disability and death at a reasonable price, the insurer would pay for it.

### Replacing Malpractice Suits

Health and life insurers would provide nearly all of the social benefits of tort law. The insurer could reward and penalize providers based on average patient outcomes. Observing poorly executed care, the insurer should demand quality improvements or end reimbursements. The insurer increases profits when it employs providers who have, or can learn, the skills required to manage high-risk cases. In under-served settings, insurers might encourage and guide expansion of primary care physicians' services. If insurers are competing for high quality services for policyholders, then the insurers should spend to retain the most skilled physicians. This should encourage physicians to hone their skills, stay current with evolving treatment, monitor their patients' outcomes and seek assistance when needed rather than risk mishaps that could threaten personal income. These penalties and rewards should address the social goals of confining clinicians' scope of practice to areas of competence and punishing low quality care.

Health and life insurers will *always *pay a price for injurious medical mistakes, whether or not mistakes come to the attention of patients, their families, or the judicial system. The insurance beneficiaries of patients who die from medical mistakes are always compensated. Young patients who die can leave fairly large settlements to their survivors by purchasing inexpensive term life policies. The life insurance benefit therefore establishes a no-fault process for compensating the survivors of patients who suffer fatal medical mistakes. After non-fatal mistakes, the insurer often will pay a new stream of health care expenses. A health and life insurance policy also could include disability insurance, comprising at least a provision to suspend premium payments in the event of disability. This would further encourage insurers to reduce non-fatal mistakes. The insurer will consistently compensate victims of low quality care, or their estates, thus addressing the third goal of tort law.

Policyholders may be willing to waive their right to jury trials if they trust health and life insurers to identify and then discourage or remediate poor quality care, encourage and refine high quality care and stand by them or compensate their survivors after adverse events. Optional independent adverse event review panels could provide arbitration and root cause analysis services.

If the health insurance premium is held constant, the savings from avoiding defensive medicine could subsidize the life insurance policy. If defensive medicine actually costs $100 billion per year in a nation of 300 million persons and could be eliminated by replacing malpractice threats with more constructive quality improvement processes, then the implied annual per capita savings could exceed $300. For healthy middle-age male non-smokers, that savings is enough to pay the entire premium for $100,000 of term life insurance. Alternatively, over 15 years the savings could pay for about $8,000 of whole life insurance. While the costs of defensive medicine are not likely to be distributed evenly across the population, savings on this scale should be available to large insurers.

If a health and life insurer can curtail unnecessary care, a much larger amount of money could be available to subsidize life insurance purchases. According to the World Health Organization Statistical Information System (WHOSIS), among western nations in 2006, the United States spent $3,100 to $4,200 more per capita on health care than Australia, Canada, France, Germany, New Zealand, Sweden, or the United Kingdom, yet US citizens faced a 109/1,000 risk of death between ages 15 and 60, 20% to 50% higher than the other nations [62]. At a state or national level, most of this money should be transferable from health care to life insurance without reducing life expectancy. Pockets of high-quality, low-cost health care in the USA convincingly demonstrate that the goal is practical [63]; what remains to be discovered is a means of consistently motivating such success. A health and life insurer in the USA should find many opportunities to profit by reducing health expenses and extending lives.

### Physician role

Health and life insurance should not reduce the practice of medicine to algorithmic care, although there is some danger of that outcome. Rather, these insurers will need to work with expert clinicians to understand as many predictable nuances of diagnosis and treatment as possible. Insurers should prize clinicians who are discriminating history takers, expert physical examiners, selective testers, accurate diagnosticians, and clever patient managers when these skills reduce costs or improve outcomes. Insurers should strive to develop and support these clinicians, as only insurers could.

## Summary

Combining health and life insurance could reconcile the health interests of patients with the financial interests of one of the best-informed but least trusted industries in health care. Negotiations between these parties could provide an objective foundation for setting the prices of many medical goods and services. One of the potential benefits is that properly implemented health and life insurance should consistently promote the social goals that malpractice lawsuits promote so inconsistently, with direct and indirect cost savings. The savings from avoiding malpractice suits and defensive medical practice could substantially offset the cost of life insurance for policyholders.

## Competing interests

The author declares that he has no competing interests.

## Pre-publication history

The pre-publication history for this paper can be accessed here:

http://www.biomedcentral.com/1472-6963/10/150/prepub
